# *Francisella* and tularemia in western Asia, Iran: a systematic review

**DOI:** 10.1016/j.nmni.2023.101092

**Published:** 2023-01-28

**Authors:** Zahra Fooladfar, Farhad Moradi

**Affiliations:** a Department of Bacteriology & Virology, School of Medicine, Shiraz University of Medical Sciences, Shiraz, Iran; b Student Research Committee, Shiraz University of Medical Sciences, Shiraz, IR, Iran

**Keywords:** Epidemiology, *francisella*, Reservoirs, Systematic review, Tularemia

## Abstract

Tularemia or rabbit fever is a transmissible disease from animals, rodents, and insects to human populations that is caused by *Francisella tularensis*. Epidemiological studies showed that tularemia is endemic throughout most different regions of the world. Recent evidence documented the transmission of the *F. tularensis* in a different part of Asia. Because there is no updated review information for tularemia in Iran, we performed this systematic review. In this study, we systematically explored biomedical databases (Google Scholar, Scopus, PubMed, and Web of sciences) to identify epidemiology, reservoirs, and carriers of Francisella in animal and human clinical specimens from 2010 to 2020, either in English or in Persian. Different studies have shown the different frequencies of F. tularensis among human and animal resources in eighteen provinces of Iran. In total, 1242 human clinical specimens, 1565 animal samples, and 355 environmental water samples were investigated to find F. tularensis in different provinces of Iran. According to the collected documents, 94 human clinical samples, 69 water samples, and 26 animal specimens were introduced as positive samples for the F. tularensis. According to studies, thirteen species of rodent and hare presented as an inter-epizootic reservoir. Only one species of tick (D. marginatus) was introduced as a vector for Francisella in Iran. According to these results, it is essential for exclusive attention to the prevalence of F. tularensis in different provinces of Iran. Furthermore, special planning should be done for prevention, control of the outbreak, and proper treatment of the tularemia.

## 1 Introduction

Bacterial zoonotic diseases are introduced as infectious agents that are naturally transmissible from vertebrate animals to humans with high mortality. The route of transmission of these agents depends on close contact with infected animals such as animal bites, arthropod vectors, animal secretion, and animal products [1]. Not only do they are endanger human health around the world but can also have a significant impact on the economies of the countries in the trade of animal food products According to many reports conducted around the world. The most famous bacterial agents that cause this type of the disease are *Bacillus anthracis* (Anthrax), and *Brucella* spp. (Brucellosis), *Listeria monocytogenes* (Listeriosis), *Salmonella* spp. (Salmonellosis), *Leptospira* (Leptospirosis), and *Campylobacter* spp. (Campylobacteriosis). However, some zoonotic agents have received less attention despite their dangerous nature in different parts of the world [1,2]. One of these microbial agents is a genus called Francisella, which is responsible for causing tularemia. According to US Centers for Disease Control and Prevention (Category A, CDC), *Francisella* is a genus of pathogenic, gram-negative coccobacillus, intracellular parasites bacteria, which are also high infectivity with low infection dose [3,4]. This species was discovered in California, in 1911 and Edward Francis introduced this bacterium as a causative agent of tularemia. The genus Francisella includes four species *F. tularensis, F. philomiragia, F. noatunensis,* and *F. hispaniensis.* In addition, *F tularensis* divided into four subspecies: *F. tularensis* subsp. tularensis, *F. tularensis* subsp. holarctica, *F. tularensis* subsp. Mediasciatica, and *F. tularensis* subsp. Novicida. This bacterium usually transferred by wild and domestic animals such as hares or rodents, handling infected animals or carcasses, during hunting and slaughtering, eating infected hares, breathing contaminated dust, eating or drinking unclean food or water, and biting from an infected tick or deer fly. However, the principal sources for humans are rodents, rabbits, and the arthropods [4,5]. In addition, signs or symptoms of tularemia vary and depend on exposure. For example, they include endotoxemia, sudden fever, chills, weakness, dry cough, diarrhea, headache, erythematous papule at the inoculation side, swollen and painful lymph glands, acute pharyngotonsillitis with a sore throat, trouble breathing and pneumonia as the most serious form of the disease that occurs three to five days after exposure [5,6]. However, clinical signs of the disease are more relevant to the type of subspecies tularensis and holarctica. The main reservoirs for subspecies tularensis (type A) are rabbits and ticks. In addition, subspecies holarctica (type B) infected aquatic animals or rodents that live near water. Type A with terrestrial and type B with water-borne cycles are responsible for almost all tularemia infections in the USA, Europe, and Asia respectively. Clinical symptoms and virulence of type A are more frequent than type B [[4], [5], [6]]. Pathogenesis of the *F. tularensis* relies on the interaction of *Francisella* ***with macrophages, dendritic cells, or neutrophils*.** Also modulated the transcription of numerous glycosidase and glycosyltransferase genes in their cells followed by the increase of N and O-protein glycosylation with significant effects on protein folding, activity, and dysfunction of protein glycosylation may lead to the development of tularemia. Within several hours following infection, macrophages can produce and release a large number of cytokines such as interferon-gamma, tumor necrosis factor-alpha, and interleukin-12. In addition, different studies demonstrate that the free-living strain of *F. tularensis* can acquire immune evasion capacity by releasing specific metabolites, which activated mucosal-associated invariant T cells in a T-cell receptor (TCR) dependent manner and alteration of metabolic programs during evolution [7,8]. *Because* *F. tularensis* known as a potential agent of bioterrorism and is transmitted by infected aerosols, to avoid laboratory-acquired infections, biosafety level three practices are required when working with the live culture of this organism. This bacterium recovers from the clinical specimens through enriched media containing cysteine and serological examinations [6], [7], [8], [10], [13]. The first animal case of tularemia in western Asia, Iran reported in 1973, and the antibody against *F. tularensis* infections was detected in different animals such as porcupines, cattle, and sheep in the southeast and northwest of Iran. Furthermore, the first human case of tularemia occurred in 1980 in Kurdistan province, Iran. Recent studies reported the frequency of *F. tularensis* in rodents and documented the prevalence of anti-francisella antibodies in the Iranian population. For instance, recently a case of tularemia was reported from Marivan city in 2017 [9]. According to different annual reports, tularemia is an endemic disease among northwest and northern neighbors of Iran such as Turkey and the Republic of Azerbaijan [11,12]. Because the recent evidence shows the transmission of the *F. tularensis* in a different part of Asia and there is no updated review information concerning tularemia in Iran, The current review study intended to investigate the status, epidemiology, reservoirs, and carriers of tularemia in animals and human clinical specimens during 2010-2022 in Iran.

## 2 Materials and methods

### 2.1 Search strategy

**Fig. 1:**
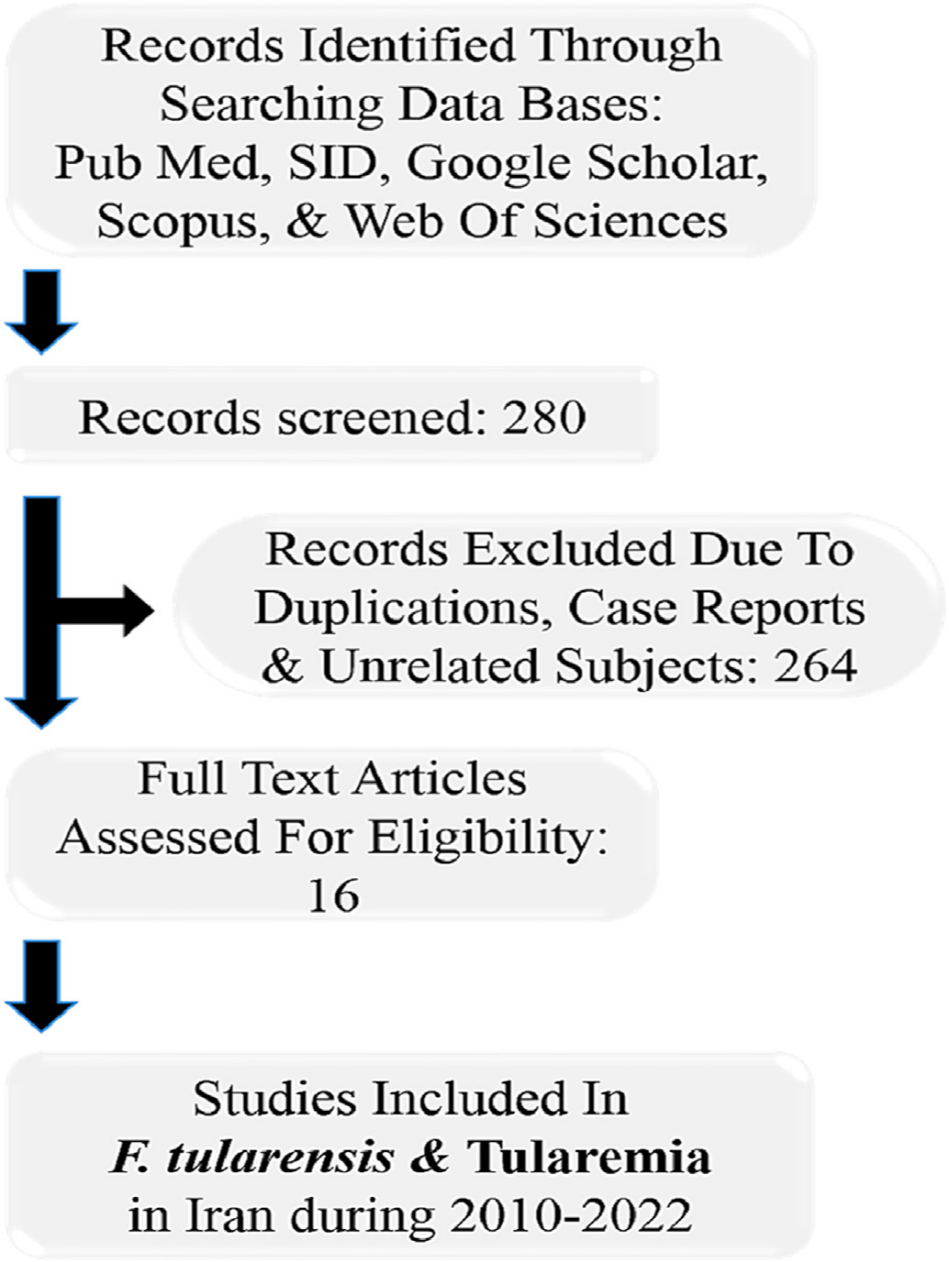
Flow chart of study.

We systematically explored biomedical databases (Google Scholar, Scopus, PubMed, and Web of sciences) to identify related studies from 2010 to 2020, either in English or in Persian. The search was performed using various combinations of the following keywords: “*Francisella* spp. AND Iran”, “*Francisella* spp. AND tularemia AND Iran”, “*Francisella* spp. AND human clinical specimens AND Iran”, “*F. tularensis* OR tularemia AND animal's reservoir AND Iran”, “*F. tularensis* OR tularemia AND food products AND Iran”, “*F. tularensis* AND epidemiology OR seroepidemiology AND Iran”. Furthermore, to increase the completeness of the exploration, extra informations were collected from the reference lists of included studies. The quality assessment was performed according to the Joanna Briggs Institute (JBI) checklist. Finally, out of 280 recognized articles, 16 papers were identified that were published between 2010 and 2022 (Fig 1).

### 2.2 Inclusion and exclusion criteria

To clarify the epidemiology and prevalence rates, we reviewed the literature that was published based on the frequency of *Francisella* in animal and human clinical specimens, reservoirs, and carriers as inclusion criteria. In addition, we considered studies that were performed in several provinces of Iran in the years between 2010 and 2020. Information about prevalence and epidemiological studies for *Francisella* and tularemia infections were characterized based on clinical human specimens, sample size, positive sample, age, gender, category of the population, and detection methods. Furthermore, the frequency in animal and small mammals such as rodents, rabbits, hares, muskrats, livestock, voles, mice, squirrels, arthropods (ticks and deer flies), and natural water samples were categorized based on the region, sample size, and type of specimens according to publication year from 2010 to 2022. Research studies that employed different detection methods such as bacteriological culture, biochemical tests, serological assessment, and molecular assays were characterized in Table 1, Table 2. Also, studies that did not explore the *F. tularensis* or tularemia, case reports studies, and duplicate documents were considered as exclusion criteria.

**Table 1:** Prevalence studies of **Francisella *and*** tularemia infections based on clinical human specimens

Authors, publication year	Performed year	Region (province)	Human specimens	Sample size No	***Category of the population (No)***	Positive sample No (%)	Gender No (%)		Age group	Detection method	References
							Male	Female			
S. Esmaeili 2014	2011-2012	Kurdistan	Blood & serum	250	Hunters (50) Butchers & Slaughterhouse (50) Health care workers (50) (100)	36 (14.4%)	205 (82%)	45 (18%)	>18	ELISA (anti-tularemia IgG)	11
S. Esmaeili 2014	2011	Sistan and Baluchistan	Blood & serum	184	Butchers & Slaughterhouse (184)	12 (6.5%)	184 (100%)	—	25-45, 3-15	ELISA (Anti-tularemia IgG)	12
A. Khoshdel 2014	2011	Chaharmahal va Bakhtiari	Blood & serum	183	Assessment among Children (11)	11 (6%)	89 (48.7%)	94 (51.3%)	2-18	ELISA-based quantitative assay	13
M. Rohani 2018	2017	Kurdistan	Blood & serum	1	A 6-year-old girl from a village Near Marivan City.	1 (100%)	_	1 (100%)	6	Serology and molecular detection (ELISA, & the ISFtu2 real-time PCR)	14
S. Esmaeili 2019	2015	Ilam	Blood & serum	367	Ranchers (112), Farmers (79), Butchers & slaughterhouse workers (61), Nature Conservation Officers (34), Referrals to Medical Diagnostic laboratories (74)	10 (2.78%)	275 (76.29%)	85 (23.71%)	>18, 18-78	ELISA	15
S. Esmaeili 2019	2017	Lorestan	Blood & serum	289	Butcher & Slaughterhouse workers (144), people from the general population (145)	11 (3.8%)	289 (100%)	_	>18	ELISA	16
H. Ahangari Cohan 2021	2018	Kurdistan	Blood & serum	51	Ranchers (51)	2 (3.92)	44 (86.27%)	7 (13.73%)	11-84	ELISA	17
S. Esmaeili 2021	2016-2018	East Azerbaijan	Blood & serum	11	patients (11) positive case for IgM/10 positive for IgG (11) positive case for tube agglutination (10)	11 (100%)	Not define	Not define	4-77	ELISA quantitative kits & Standard tube agglutination	18

**Table 2:** Prevalence studies of *Francisella* species. Based on animal and water specimens

Authors, publication year	Performed year	Region (province)	Animal, food & water sample	Type of specimens	Sample size No	Positive sample No (%)	Method	Reference
B. Pourhossein 2015	2013	Sistan va Baluchestan	Animal (rodent With 48 fleas & 10 ticks)	Blood collection from the hearts of the rodents.	9	1 (11.1%) )with serum titers of 1/80 (	Serology method	19
E. Mostafavi 2016	2015	Kurdistan	Animal (rodents)	Blood & spleen tissue	245	12 (4.8%)	Standard tube agglutination assay for tularemia & Bacterial culture, Microscopic examination, & Real-time PCR	20
E. Mostafavi 2018	2014-2015	Northern Khorasan, Khorasan Razavi, Fars, Golestan, Zanjan, Chaharmahal va Bakhtiari, Semnan, Sistan va Baluchistan, Khuzestan, Kerman and Kermanshah	Animal (140 rodents, 17 insectivores, & 51 hares)	Spleen tissue	208	5 (2.4%) (Three of 140 rodents, Two of 51 hares)	Real-time PCR assays	21
M.Rohani 2019	2015	Kurdistan, Western Azerbaijan	Natural water	Natural water	237	52 (21.94%)	Cultured and the ISFtu2 real-time PCR	22
M.Hemati 2020	2014-2017	Hamadan province	Animal sample (rodents)	Blood sample, spleen tissue	407 serum sample, 433 spleen sample	3 (0.74%) for serological test & 5 (1.15%) for molecular test	Serological test & Real-time PCR	23
H. Ahangari Cohan 2020	2018	Kurdistan province	surface water	surface water	66	3 (4.54%) positive for *ISFtu2/*1 (1.51%) positive for *fopA* genes.	Targeting ISFtu2 and fopA genes using TaqMan real-time PCR.	24
H. Ahangari Cohan 2021	2018	Kurdistan province	Animal (289 sheep &103 cattle)	Blood sample, Spleen sample	392	0 positive for agglutination titr/0 positive for *ISFtu2* gene nor for *fopA* gene	standard tube agglutination method & real-time PCR	17
S.Esmaeili 2021	2016-2018	East Azerbaijan	Animal (rodents)	Blood sample/Spleen sample	9	One out of nine captured rodents positive for ISFtu2 elements and fopA gene/no positive for culture and serological testing	Real-time PCR/Standard tube agglutination/culture	18
S.Esmaeili 2021	2016-2018	East Azerbaijan	surface water	surface water	6	1 positive for ISFtu2 elements and fopA gene.	Cultured, Real time PCR	24
Sh. Aghamohammad 2022	2019	East Azerbaijan	Water,	Water sampling & internal organs of mice that inoculate with water sample	46	1/46 (2.2%) positive for ISFtu2 gene from molecular analysis of the pellets obtained from washed filters, 10/46, (21.7%) positive from mice tissues, one positive sample with both ISFtu2 and fopA genes in real-time PCR	Cultured, TaqMan real-time PCR method	25
M.Rahravani 2022	2020-2021	Kurdistan province	Animal sample (small ruminants, sheep& goats)	Blood sample	250 Sheep 232 (92.8%), Goats 18 (7.2%)	With No positive result	The Real Time-TaqMan PCR (ISftu2 gene)	26
			Animal sample (ticks)	salivary gland & sexual organ tissue	244	2 (0.8%)		

### 2.3 Data analysis

In this review, two researchers refute any possibility of error accomplished in data extraction. Statistical analyses such as data storage, numerical, average calculating, and chart designing were performed using Microsoft EXCEL 2022.

## 3 Results

### 3.1 Epidemiological information for pathogenic *F. tularensis*

**Fig. 2:**
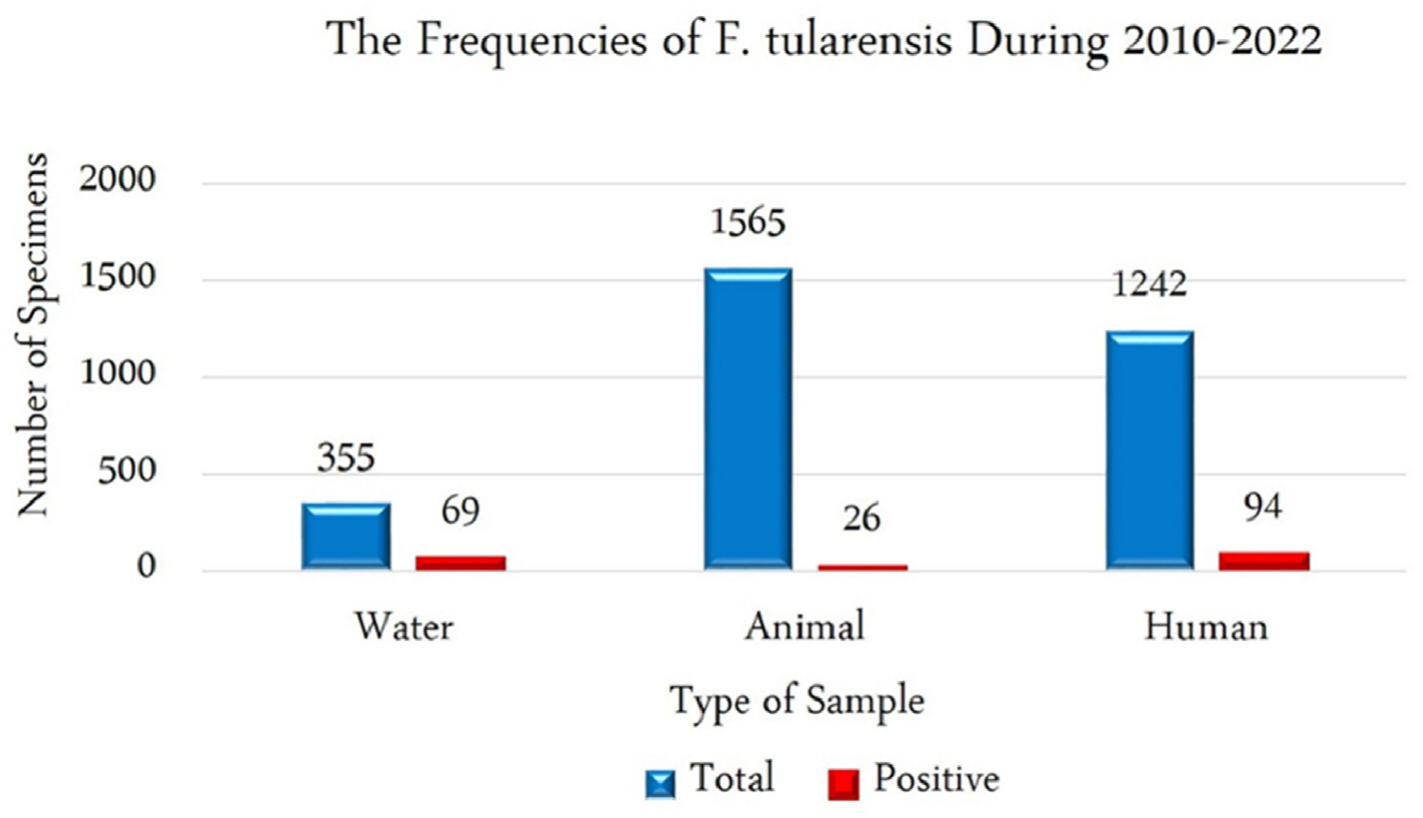
the frequency of the *Francisella tularensis* in Iran during 2010 - 2022.

**Fig. 3:**
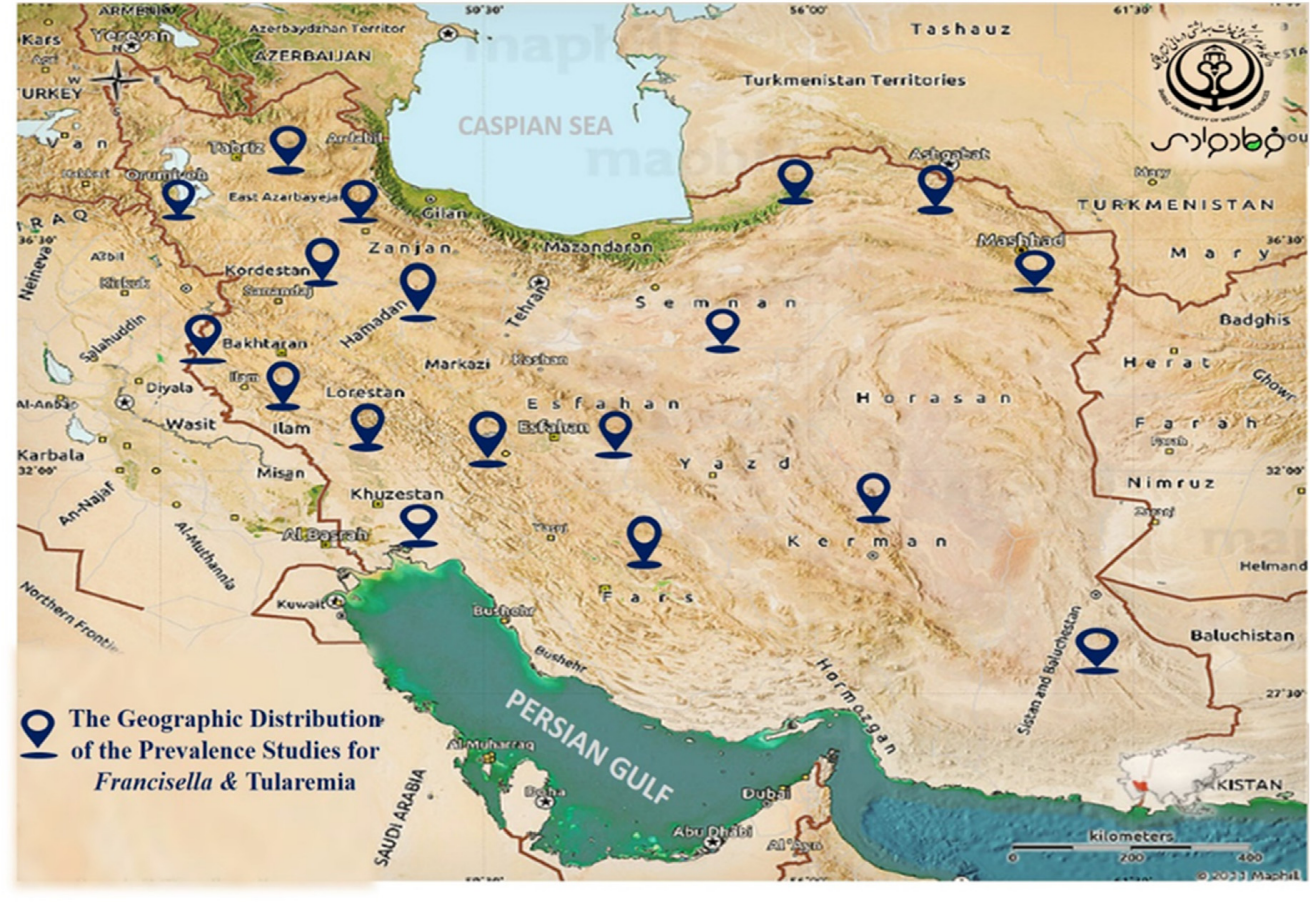
The geographic distribution of the epidemiological studies for *Francisella tularensis* in different provinces of Iran.

The frequency information of the *F. tularensis* and tularemia in Iran were characterized and accessible in Table 1, Table 2, Table 3. In total, 1242 human clinical specimens, 1565 animal samples, and 355 environmental water samples were investigated to find *F. tularensis* in different provinces of Iran during 2010-2022. According to the collected documents, 94 human clinical samples, 69 water samples, and 26 animal specimens were introduced as positive samples for the *F. tularensis* (Fig 2). Epidemiology records about the incidence of *F. tularensis* among animal specimens were categorized according to genus and species of rodents, fleas, mites, ticks, hares, and insectivores (Table 3). Most studies had been performed during 2011–2013 (for clinical human specimens (n = 3)) and during 2015-2018 (for animal and water specimens (n = 7)). We selected four observational cohort studies that were performed in different provinces. Furthermore, these studies were completed in eighteen provinces such as Fars, Chaharmahal va Bakhtiari, Lorestan, Kurdistan, east Azerbaijan, Sistan va Baluchistan, and Golestan. However, most studies were performed in two provinces of Kurdistan and Azerbaijan. According to different reports, most of the *F. tularensis* infections were discovered in Kurdistan, Sistan va Baluchistan, and Chaharmahal va Bakhtiari provinces based on clinical assays among human populations. Moreover, in Kurdistan and western Azerbaijan provinces among animals and water specimens. The geographic distribution of the epidemiological studies in different provinces on a map of Iran is shown in Fig 3.

**Table 3:** Epidemiology of *F. tularensis* among animal specimens according to genus and species

Authors, publication year, reference							
Animal samples		S. Esmaeili 2021, 18	B. Pourhossein 2015. 19	E. Mostafavi 2016., 20	Ehsan mostafavi 2018, 21	M.Hemati 2020, 23	M.Rahravani 2022, 26
Genus and species of	Rodents (NO)	9 sample collected: *Microtus socialis (1),* *Microtus socialis (2),* *Microtus mystacinus(1),* *Dryomys nitedula (1),* *Apodemus witherbyi (2),* *Arvicola persicus (1)* *Meriones persicus (1).*	9 sample collected: *Tatera indica* (Indian gerbil)	245 sample collected; most of them contained *Apodemus* (*Apodemus witherbyi,* 40%) *Mus* (*Mus macedonicus*2, 4.5%) *Meriones* (*M. persicus,* 12.6%)	140 sample rodents the most common: *30 Microtus paradoxus* (21%), 17 *Apodemus witherbyi* (12%), 16 *Microtus irani* (11%), 15 *Mus musculus* (11%), 14 *Microtus socialis* (10%).	433 sample collected: *Meriones persicus (327), Meriones libycus(44), Meriones vinogradovi (24), Ellobius lutescens (13), Microtus qazvinensis (8), Spermophilus fulvus (7), Meriones tristrami (5) Arvicola persicus (2), Calomyscus elburzensis Isatissus(2), Mus musculus domesticus* (1)	—
	Fleas (NO)	*-*	48 fleas: *Xenopsylla* spp	153 fleas: *X. buxtoni* (123), *Paraceras melis melis* (11), *Ctenophthalmus iranus persicus* (11), *Leptopsylla segnis* (6),*Parodoxopsyllus microphtalmus* (1), *Ctenophthalmus rettigi smiti* (1)	—	—	—
	Mite (NO)	—	—	37 mites: *Eulaelaps stabularis* (15), *Haemolaeps glasgowi* (13*), Laelaps nuttalli* (6), *Echinolaelaps echidninus* (2), *Dermanyssus sanguineous* (1).	—	—	—
	Ticks (NO)	—	10 ticks: *Hyalomma* genus	54 ticks: *Haemaphysalis spp*. (35), *Hyalomma spp.* (19).	—	—	244 ticks collected: *Dermacentor marginatus* (164), *Rhipicephalus turanicus* (30), *Rhipicephalus sanguineus* (26), *Haemaphysalis concinna* (24)
	Hares (NO)	—		—	51 hares were collected: 29 *Lepus europaeus* (57%), 7 *Lepus tolai* (14%) and 15 *Lepus sp*. (29%)	—	—
	Insectivores (NO)	—	—	—	17 insectivores were collected: 14 *Crocidura suaveolens* (82%), 3 *C. leucodo* (18%)	—	—
Result		One *Microtus socialis* out of nine captured rodents was positive for ISFtu2 elements and fopA gene.	serum agglutination test was positive for tularemia in one of the rodents	Serological tests were positive for tularemia in 4.8% of trapped rodents.	Three rodents (*Apodemus uralensis, Musmusculus domesticus*, and *Chionomys nivalis*) and two hares (*L. europaeus* and a *Lepus sp*.) were positive for *F. tularensis*.	8 cases were positive (*M. persicus* = 6, one *M. libycus = 1*, and *M. vinogradovi* = 1)	2 tick samples was positive Both ticks were classified as male D. marginatus

### 3.2 Prevalence rate and detection method

**Fig. 4:**
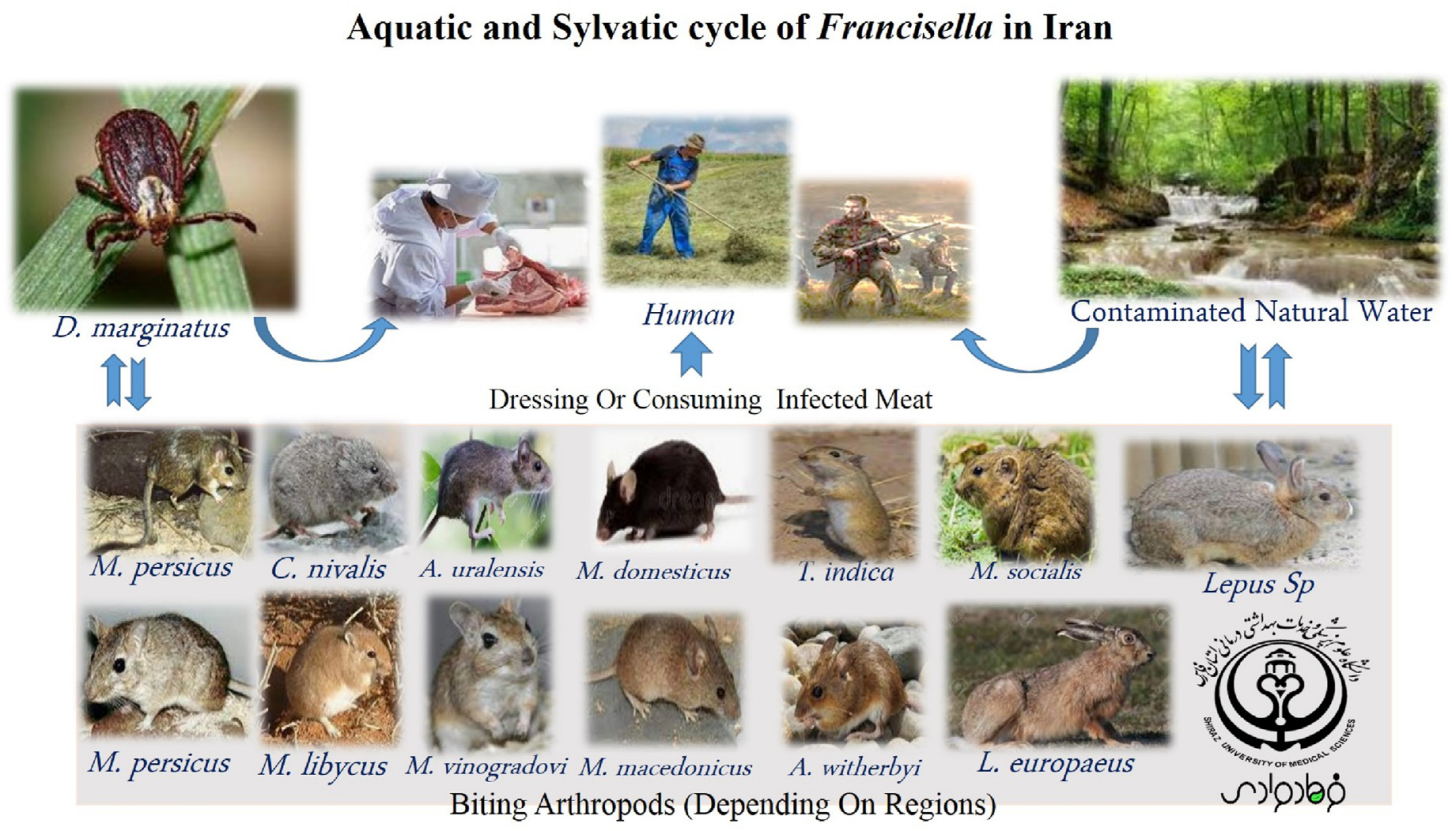
Possible aquatic and sylvatic cycles of *Francisella* in Iran.

According to performed studies related to human societies, blood and serum specimens are considered as the most important sample for the investigation of *F. tularensis.* Many of these specimens were isolated from adult and pediatric groups with different signs and symptoms. Sero-epidemiological studies were performed based on different serological tests such as standard tube agglutination, rapid test VIRAPID® TULAREMIA strip (Vircell, Spain), and ELISA quantitative assay (anti-tularemia IgG). In addition, some of the studies performed by molecular method such as PCR or Real-time PCR assay. Human studies performed among various human groups including hunters, butchers, slaughterhouse, health care workers, ranchers, farmers, nature conservation officers, and medical diagnostic laboratories staffs in six provinces (Table 1). However, we selected other studies conducted in Iran from 2010 to 2022 about the occurrence of Francisella among the animal population as a reservoir including rodents, insectivores, hares, sheep, cattle, and goats. In addition, different genera and species of insects that were isolated from these animals including fleas, mites, and ticks, were investigated for the presence of Francisella. Most of the collected specimens in these studies involved blood samples, spleen tissue, salivary gland, and sexual organs tissue. In addition, the genus and species of the animals showed in detail in Table 3. Besides, in several provinces such as Kurdistan, Western, and East Azerbaijan, surface and natural water samples were analyzed for contamination with *Francisella*. All of the isolates were assessed for *Francisella* or tularemia through microbiological methods such as microscopic examination, culture, and serological tests. Furthermore, molecular assay was performed by Real Time-Taq Man PCR and specific primers for different genes such as *tul 4* (encoded Lipoprotein, T-cell-stimulating antigen)*, ISFtu2* (insertion sequences elements 2)*,* and *fopA* encoded a heat-modifiable outer membrane-associated protein that required for the growth of *F. tularensis* murine macrophages genes. According to studies, thirteen genera and species of rodent and hare, and only one species of tick were introduced as a positive sample for *Francisella* infection. Many of these species contain *M. persicus*, *C. nivalis, A. uralensis, M. domesticus, T. indica, M. socialis, M. persicus, M. libycus, M. vinogradovi, M. macedonicus, A. witherbyi, L. europaeus, Lepus* sp., and *D. marginatus.* Additionally, according to the studies probable aquatic and sylvatic cycles of *Francisella* in Iran were designed in Fig 4.

## 4 Discussion

Tularemia or rabbit fever is a transmissible disease from animals and insects such as rodents, rabbits, hares, ticks, and deer flies to humans that is caused by *F. tularensis.* Recently, it has been proven that tularemia may appear as an emerging disease in areas where the disease has not been reported for many years. Epidemiological studies showed that tularemia is endemic and diverse throughout most of Europe, Asia (type B), North America (type B and type A), and Russia. This issue may due to the variety of natural recourse, animals, and people [15], [16], [17], [27], [28]. This bacterial species can survive for several weeks in the environment. Their infections diagnosed by clinicians based on symptoms, history, and laboratory studies of the patients. Iran is a vast country in Western Asia that has a high variety of genera and species of animals and rodents, as well as natural waters. Considering the clinical importance of *Francisella* and the presence of these natural resources in Iran, it seems necessary to study and monitor this bacterium in different provinces. Some interesting results are mentioned based on the studies conducted in connection with the epidemiology of the disease among human populations. For instance, different countries such as France, Spain, Slovakia, the Czech Republic, and Germany introduced hare hunting as the main cause of glandular infections [19], [20], [21], [22], [23], [28], [29]. Although serological and molecular studies conducted among different human societies in Iran show that some participants have specific clinical symptoms of tularemia, a large number of studies have shown that they are asymptomatic. For instance, clinical manifestations included mild lymph adenopathy of the axillary lymph nodes followed by consuming the meat of a hunted hare, the development of swollen lymph nodes in the neck of patients living within an area with previous reports of tularemia with fever, fatigue, and muscle aches. Furthermore, redness of the oropharyngeal mucosa, and typical oropharyngeal tularemia through drinking contaminated water or eating food washed with contaminated water [14], [18], [24]. Although serological techniques such as ELISA tests, micro agglutination tests, and indirect immunofluorescence assays have weak reliability and standard, they are very applicable to epidemiological studies. However, molecular methods such as PCR or Real-time PCR are reliable for the detection of *F. tularensis* in clinical specimens from humans, animals, and water samples because they can identify viable but non-cultural state (VBNC) state or symbiotic form within the amoeba in aqueous environments. In addition, these methods have been used for the primary isolation of the organism from blood samples, spleen tissue, and lymph nodes in different rodents, insectivores, hares, and even from fleas or ticks [25], [26], [30], [31]. Many studies conducted in Iran have used serological and molecular methods to diagnose and confirm Francisella infections at the same time. For example, in 2017, these methods were performed to detect this bacterium in a six-year-old child in Kurdistan province [14]. In addition, tularemia was introduced as a vector-borne disease, and reports considered different ticks species such as *O. parkeri, O. hermsi, O. moubata, D. andersoni,* and *D. parumapertus* as the main natural vector or reservoirs for *F. tularensis.* Although ticks or mosquitoes are not considered a common cause of tularemia infection in Scandinavia, Europe, and Sweden, Iranian research studies considered various tick species as the main vector for *F. tularensis* in parallel to North America and Eurasia [28,32,33]. D. marginatus has incriminated as relevant to the enzootic cycle of tularemia in Iran (Fig 4). In different regions of the world, tularemia suggests as a non-rodent or non-vector-associated infection acquired from hunting, agriculture, threshing, and natural waters contaminated by the presence of animals and their excrement. Besides, the transmission cycle of Francisella is very different in different parts of the geographic area. For instance, the tularemia outbreak in Castilla y Leon, Spain, American, Soviet, and Swedish workers recognized by harvest activities. Furthermore, water-borne tularemia was first described by Karpoff and Antonoff in their description of an outbreak related to drinking river water and similar to these reports from Eurasia. In addition, the rate of contamination with *F. tularensis* in water sources in Sweden and Utah USA has been reported at 32% and 39% cases respectively. On the other hand, Francisella has been isolated from environmental waters in different provinces of Iran, including Kurdistan, and Western and East Azerbaijan provinces. *F. tularensis* subsp. holarctica has been isolated in North America, Japan, Asia, and Europe, and is associated with waterborne infection in rodents, and different vectors [29,[33], [34], [35], [36], [37], [38]]. Although *F. tularensis* cause lymphadenopathy, pinpoint white spots on the liver and spleen, chronic nephritis, bacteriuria, and necrotic manifestations among rodents, they are known as a putative reservoir or vector for the transmission cycle of this bacterium. For instance, the outbreak of 2007–2018 and a lesser one in 2014 coincided with irruptions of common voles, Microtus arvalis in Castilla y León [39,40]. In addition, *F. tularensis* is present in East Kazakhstan by periodic epizootics in rodents or Spain large pneumonic outbreak was associated with direct contact with common voles, Microtus arvalis [41,42]. According to different reports in Iran, the epidemiological situation of the *F. tularensis* was introduced by the role of different rodents and hares that known as a vector and interepizootic reservoir. Besides, transmitted Francisella among these rodents through the contamination of environmental water sources with animal waste, aerosol, or the tick, and risk to people was associated with biting arthropods, contamination of drinking water, dressing or consuming infected meat, tick bites, and agriculture. Finally, frequency among hunters, butchers, health care workers, ranchers, farmers, nature conservation officers, and nature conservation officers. In Iran, the highest prevalence of tularemia is observed among hunters, butchers, health workers and probably type B is the most common subtype of tularemia in Iran. Because less attention has been paid to tularemia in the training programs of physician in Iran and lack of general laboratory diagnostic facilities until the last few years, the limitation of reporting of patients that suffering from tularemia can be justified to some extent [42,43]. Hence, it is necessary for exclusive attention and follow-up of infection in different regions, especially in rural areas. In addition, there is a need for future studies to separate research for current sources such as water, and wild rodents and identify the common type of *F. tularensis* in Iran. Consequently, Iranian physicians and health workers must gain the necessary knowledge about the circulation of this bacterium in different regions of the country so this tularemia considered for differential diagnosis at least in the minds of doctors.

## 5 Conclusion

Success in preventing human tularemia requires the prevention of contact with the carrier of these organisms and infected animal tissue. The most effective prevention methods include teaching people to protect themselves against mosquito, tick bites and avoiding drinking or swimming in contaminated water in areas where the infection is common among wild animals. Children, who live in endemic areas where ticks are infected with bacteria should regularly check their skin for the presence of ticks and, if present, remove them with tweezers. When pets die due to this disease, they are completely buried in a suitable place. Rabbit hunters must be fully aware of the ways of transmission and clinical symptoms of the disease, and if laboratory workers exposed to effective contacts such as centrifuge accidents or being hit by a needle head infected with samples containing *Francisella*, it is necessary to cover by post-contact treatment.

## Author contributions

Farhad Moradi: Conceptualization, Methodology, Software, Supervision, Writing- Reviewing and Editing. **Zahra Fooladfar**: Data curation, Writing- Original draft preparation, Investigation and Validation.

## Data availability

Data availability We thanks Department of Bacteriology and Virology, School of Medicine, Shiraz University of Medical Sciences (SUMS) for scientific assistance in carrying out this study.

## Funding/support

There is no funding or support.

## Source of support

None.

## Conflicting interest

None.
